# Reduced Taurine Synthesis Underlies Morphine-Promoted Lung Metastasis of Triple-Negative Breast Cancer

**DOI:** 10.3390/cancers17071086

**Published:** 2025-03-24

**Authors:** Shih-Hong Chen, Ting-Ling Ke, Chien-Hung Shih, Chia-Ni Hsiung, Kuo-Chin Chen, Zi-Xuan Huang, Tsung-Hsien Chuang, Li-Kuei Chen, Linyi Chen

**Affiliations:** 1 Institute of Molecular Medicine, National Tsing Hua University, Hsinchu 300044, Taiwan; pcegg2002@gmail.com (S.-H.C.); happy1425tw@gmail.com (T.-L.K.);; 2 Department of Anesthesiology, Cathay General Hospital Medical Center, Taipei City 106438, Taiwan; 3 Immunology Research Center, National Health Research Institutes, Miaoli City 350401, Taiwan; 4 College of Medicine, China Medical University, Taichung City 406040, Taiwan; 5 Department of Anesthesiology, China Medical University Hospital, Taichung City 404327, Taiwan; 6 Department of Medical Science, National Tsing Hua University, Hsinchu 300044, Taiwan

**Keywords:** cysteine dioxygenase type, glutamate decarboxylase 1, morphine usage, taurine biosynthesis, triple-negative breast cancer

## Abstract

**Background**: The mechanisms underlying the progression and metastasis of triple-negative breast cancer (TNBC) in the context of extended morphine exposure remain poorly understood. Morphine consumption has been a pressing issue in many countries. While the physiological impact of extended morphine use is multifaceted, cancer patients with a history of extended morphine usage often have a poor prognosis. **Methods**: In this study, we investigated the impact of extended morphine treatment on the transcriptional profiles of TNBC. To this end, mice were administered morphine intraperitoneally for 14 days, followed by the implantation of EO771 cells, which are triple-negative breast cancer cells, into their mammary fat pad. After primary tumors were removed on the 38th day, a subset of mice was continuously given saline or morphine until the 68th day. Tumor size, organ metastasis, and tumor RNA expression were analyzed. **Results**: Our findings showed that extended exposure to morphine led to an increase in lung metastasis in the mouse model of triple-negative breast cancer. We analyzed RNA sequencing on tumors to compare their transcriptional profiles with or without metastasis. Through pathway analysis, we specifically examined the novel impact of morphine on the downregulation of taurine/hypotaurine biosynthesis. Given that morphine, droperidol (a dopamine receptor antagonist), and naloxone (an opioid receptor antagonist) might act through either opioid receptors or dopamine receptors, we further demonstrated that taurine mitigated EO771 cell invasion induced by morphine but not by droperidol or naloxone treatment. Additionally, morphine treatment markedly decreased the expression of *GAD1*, one of the enzymes essential for taurine biosynthesis, whereas droperidol and naloxone did not. **Conclusions**: The findings of morphine-induced reduction in *GAD1* levels and the inhibition of invasion by taurine treatment suggest that taurine could serve as a potential supplement for triple-negative breast cancer patients who require morphine as part of their treatment regimen or due to their circumstances.

## Simple Summary

Morphine is commonly prescribed for managing severe pain; however, its long-term effects on the progression and metastasis of triple-negative breast cancer remain unclear. In this study, we investigated whether extended morphine exposure influences tumor growth and lung metastasis in an animal model. Gene expression analysis revealed that morphine downregulated *GAD1*, which encodes an enzyme essential for taurine synthesis—an amino acid known to counteract invasive cancer cell behavior. These findings suggest that taurine supplementation may be a potential strategy for patients with triple-negative breast cancer undergoing extended morphine treatment.

## 1. Introduction

As morphine is commonly used for pain treatment, it is also used to treat various diseases, such as coronary artery disease, pulmonary edema, and acquired immunodeficiency syndrome (AIDS) [1,2]. However, except for analgesic effects, morphine also modulates the immune system, which could have an influence on tumor progression. Chronic usage of >120 mg/day in clinical settings may lead to opioid tolerance or addiction [3]. Breast cancer patients might have been prescribed morphine for an extended duration for reasons such as myofascial pain syndrome or chronic pancreatitis before encountering cancer-related pain. Consequently, they face a considerable risk of tumor progression due to immunosuppression [4,5]. Such morphine usage is a particular concern among patients who require a higher morphine dosage before a cancer diagnosis is made.

Breast cancer, especially triple-negative breast cancer (TNBC), is a leading cause of cancer-related death in women due to its heterogeneous characteristics and lack of target therapies [6]. Patients diagnosed with TNBC usually bear severe cancer pain, and they require an increasing morphine dosage as the disease progresses [7]. It is an important issue to investigate whether extended morphine usage could inadvertently exacerbate tumor growth or metastasis.

The mechanisms underlying morphine-mediated tumor growth and metastasis remain unclear. Mathew et al. reported that knockout of the μ-opioid receptor (MOR) or using a MOR antagonist considerably inhibited tumor growth [8]. Proposed mechanisms include the release of substance P, the upregulation of Survivin expression, and c-Jun N-terminal kinase-mediated activation of the mitochondria-dependent pathway [9,10,11]. On the other hand, morphine has been reported to decrease metastasis and invasion by reducing metalloproteinase activity and weakening endothelial cell adhesion molecule [12,13]. Nonetheless, the clinical data point to a more significant potential of morphine to promote, rather than inhibit, metastasis [12,14,15]. The discrepancy in the results may lie in the duration of morphine usage. These alterations may create a permissive tumor microenvironment (TME) for cancer progression, especially TNBC. Some studies have reported taurine, an abundant free amino acid derived from cysteine metabolism, as a potential modulator of TME [16]. In TME, lactate accumulation of lactate and reduced glucose levels lead to acidification, which promotes cancer invasion and metastasis. Taurine counteracts the acidic and nutrient-derived microenvironment that results from the Warburg effect in rapidly proliferating cancer cells [17].

Recent studies have reported potential links between morphine usage and tumor progression, although conflicting data suggest both pro- and anti-tumor effects under different experimental settings [18]. For TNBC patients, morphine may be associated with an enhanced metastatic trend, potentially mediated through immunosuppression and direct effects on cancer cell proliferation [7,19]. The real mechanism is still obscure. Given TNBC’s invasive ability and the prevalence of morphine usage among patients, it is necessary to clarify how extended morphine exposure influences TNBC progression and is important to optimize both pain treatment and patient outcomes. Morphine is an effective drug for treating severe breast cancer-related pain symptoms. The morphine dosage needs to be increased as pain progresses due to tolerance. Recent studies have reported the adverse effect of morphine on tumor growth and metastasis, including in triple-negative breast cancer [20,21]. Triple-negative breast cancer is characterized by low expression levels of the estrogen receptor, progesterone receptor, and human epidermal growth factor receptor and accounts for 10–20% of all breast cancers. The rate of metastasis is higher in triple-negative breast cancer than in other breast cancer types, and the survival probability in triple-negative breast cancer is thus lower due to the lack of a therapeutic target.

Most existing research focuses on morphine administered for immediate pain relief, with less attention on the effects of extended morphine use or pre-exposure to morphine. For this reason, studying the impact of extended morphine usage beyond its conventional role as a painkiller during cancer treatment shall provide a unique perspective regarding the effects of morphine on cancer progression. The present study aims to compare the gene expression profiles of metastatic tumors induced by extended morphine exposure to those of spontaneous triple-negative breast tumors.

## 2. Methods and Materials

### 2.1. Reagents

RPMI-1640, fetal bovine serum (FBS), and 6-diamidino-2-phenylindole were purchased from Invitrogen (Carlsbad, CA, USA). Additionally, 3-(4,5 dimethylthiazol-2-tl)-2,3-diphenyltetrazolium bromide (MTT) was purchased from Sigma-Aldrich (Saint Louis, MO, USA). Bovine serum albumin (BSA) was purchased from Santa Cruz Biotechnology (Santa Cruz, CA, USA). The SPLInser polyethylene terephthalate membrane (pore size: 8.0 μm) was obtained from SPL Lifesciences (Pocheon, Republic of Korea). Matrigel was purchased from BD Biosciences (San Jose, CA, USA). TRIzol reagent was purchased from Invitrogen. The SYBR green master mix, reverse transcription kit, and StepOnePlus PCR system were purchased from Applied Biosystems (Foster City, CA, USA). Taurine was from Sigma-Aldrich (Saint Louis, MO, USA), and morphine sulfate was from Taiwan Food and Drug Administration, Ministry of Health and Welfare, Taiwan. Droperidol was purchased from the Excelsior Pharmatech Labs (Taipei, Taiwan), and naloxone was purchased from UniPharma (Taipei, Taiwan).

### 2.2. Cell Culture, Animal Handling, and Ethics Statement

EO771 cells [22,23,24] (obtained from Dr. Tsung-Hsien Chuang, National Health Research Institutes, Taiwan) were maintained in six-well plates with RPMI-1640 supplemented with 10% FBS at 37 °C and 10% CO_2_. Female C57BL/6 mice aged 12–14 weeks were purchased from the National Laboratory Animal Center, Taiwan, and housed in a 12/12 h light/dark cycle.

**Figure 1:**
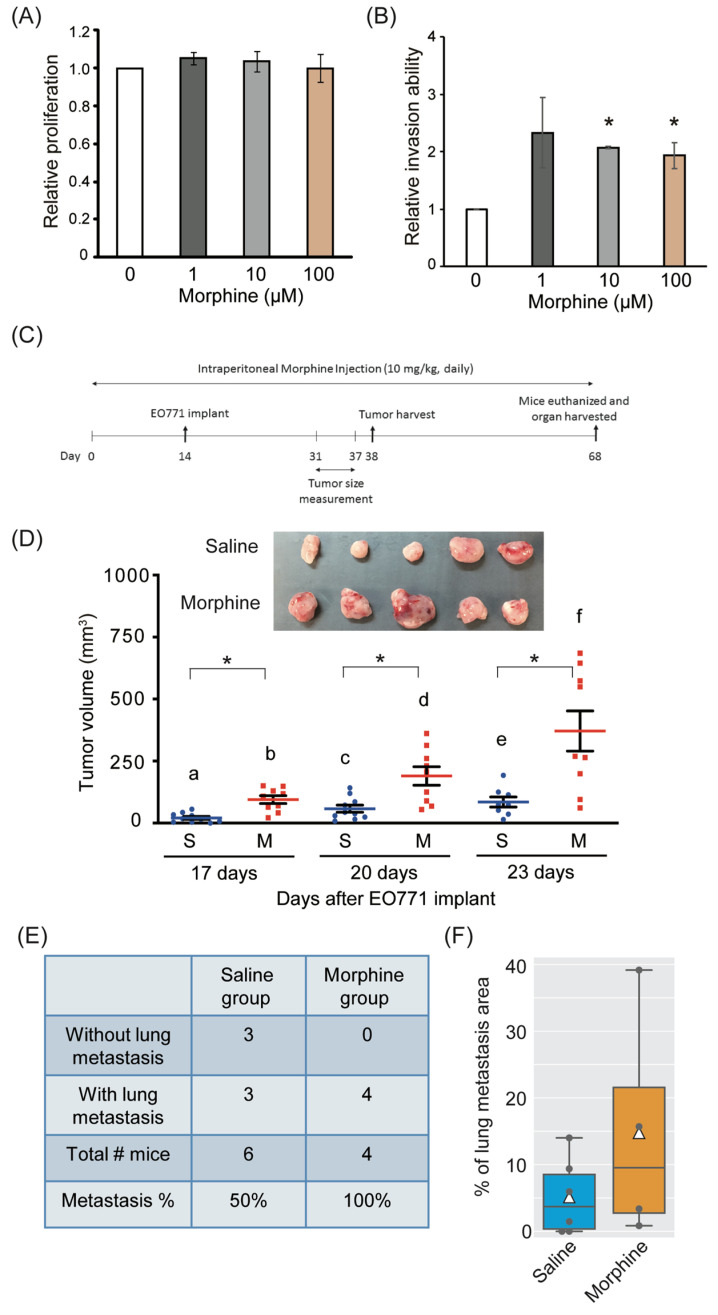
Schematic of the experiments and the effect of morphine on the proliferation and invasion of EO771 cells. (**A**) EO771 cells were treated with 0–100 μM morphine. MTT assays were performed to determine relative cell proliferation after 48 h of treatment. (**B**) EO771 cells were treated with 0–100 μM morphine for 26 h, and the Boyden chamber assays were performed to determine relative invasion ability (* indicates a significant difference of *p* < 0.05 between 0 and morphine-treated groups, determined using Student’s *t*-test). Data were from three independent experiments. (**C**) Timeline of the study. The mice received an intraperitoneal morphine injection for 14 days. On day 14, EO771 cells were implanted into a fat pad. Tumor size was measured from day 31 to day 37. Primary tumors were collected on day 38; this was followed by suturing of the wound area. Based on the health condition of the mice, 6 saline-treated and 4 morphine-treated mice were continuously administered saline or morphine till day 68. Liver and lung samples were collected on day 68 to determine possible metastasis. (**D**) Tumor resection 23 days after tumor implant. Tumor size data and images of saline-administered mice were previously published. Adapted with permission from Ref. [28] (Licence number 5770211109634), 2022, Springer Nature. Tumor volume (mm^3^) was measured on the 17th, 20th, and 23rd day after EO771 implantation. The original number of animals was 11 in the saline group and 10 in the morphine group. In the saline (S) group, the number of animals analyzed on the 17th, 20th, and 23rd day was 10, 10, and 8, respectively. In the morphine (M) group, the number of animals analyzed on the 17th, 20th, and 23rd day was consistently 9. Outliers exceeding the mean ± 1.5 standard deviations were excluded from the analysis. Values with different letters (a~f) indicate statistically significant differences (two-way ANOVA, *p* < 0.05), and the “*” symbol indicates a statistically significant difference (Student’s *t*-test, *p* < 0.05). (**E**) Metastasis % was calculated based on the number of mice with lung metastasis in saline-treated and morphine-treated mice. (**F**) The % of lung metastasis area in saline-treated and morphine-treated mice was calculated as the ratio of the metastatic tumor area to the total lung area for each sample. No significant difference was noted (Student’s *t*-test was used for statistical analysis, *p* = 0.357).

Experimental mice (*n* = 11, C57BL/6Jarl, female) were intraperitoneally injected with morphine (10 mg/kg/day) or an equal volume of saline for the indicated days (control group, *n* = 10) (Figure 1C). In previous studies, a dose of 10 mg/kg has been commonly used in experiments involving mice [25]. We selected this dosage to pre-treat with morphine to simulate real-world scenarios where many patients have already been using morphine long-term for various reasons prior to cancer diagnosis. Previous research has demonstrated that mice develop analgesic tolerance 14 days after morphine administration [26], making this a suitable model for mimicking clinical conditions in patients with chronic morphine use. The dosage used in mice is approximately equivalent to a human dose of 2.4 mg/kg [27]. The EO771 cells were implanted into the mammary fat pad (2 × 10^5^ cells in 150 μL of PBS) on the 14th day. A tail-flick test was performed 1 h after 10 mg/kg morphine injection to evaluate delayed nociception to a heat stimulus. Tumor size was measured every 3 days from the 31st day. Thirty-eight days after the first morphine injection, the tumor was resected under general anesthesia (1.5% isoflurane with oxygen). After removing the primary tumors, six mice from the saline-treated group and four from the morphine-treated group were continuously given saline or morphine and euthanized on the 68th day. Their livers and lungs were also harvested to evaluate metastasis on day 68. All animal experiments were conducted according to guidelines of the Laboratory Animal Center of National Tsing Hua University (NTHU), Taiwan. Animal usage protocols were reviewed and approved by NTHU’s Institutional Animal Care and Use Committee (approval number: 10607, approved on 22 March 2017).

### 2.3. Cell Proliferation and Invasion Assays

The EO771 cells used for proliferation and invasion assays were from the same source and passage number. Triplicates were used for each independent experiment. At least three independent experiments were performed. To determine the effect of morphine on cell viability/proliferation, EO771 cells cultured in a 96-well plate were treated with different morphine concentrations (1, 10, or 100 μM) for 48 h. MTT assays were performed to determine relative cell viability (co-incubated time 3.5 h, and concentration 0.5 mg/mL). Morphine stock solution was serial-diluted with RPMI medium (without FBS); thus, RPMI was added to the culture as the negative control (0 morphine).

To determine cell invasion, Boyden chamber assays were performed to analyze cell invasion ability. A transparent polyethylene terephthalate (PET) membrane (8 μm) insert was coated with Matrigel in 24-well plates, and the EO771 cells were suspended in RPMI (containing 0.1% *w*/*v* BSA, 10^6^ cells/mL) and plated onto solidified Matrigel. RPMI medium (supplemented with 10% FBS) was added to the lower chamber as a chemoattractant to attract cell invasion. After incubation for 26 h, the cells migrated to the bottom side of the PET membrane and were fixed with 4% paraformaldehyde and stained with crystal violet. Images of crystal violet-stained cells were taken using a Zeiss Observer Z1 microscope (Zeiss, Jena, Germany). The cells were counted using Image J software version 1.54 g (https://imagej.net/ij/index.html) (accessed on 21 May 2022). Relative invasion ability was calculated as cell number per chamber and normalized to “0 morphine” control (=1).

To calculate the relative lung metastasis area, we calculated the ratio of the metastatic tumor area to the total lung area for each sample. We accounted for variations in lung size between samples to ensure that the metastasis measurement accurately reflected the proportion of the lung area affected, rather than the absolute size of the metastasis. The formula used is as follows:

Percentage of lung metastasis area = (Metastatic tumor area/Lung area) × 100%

### 2.4. Real-Time Quantitative PCR

RNAs of the EO771 cells were collected using TRIzol, and mRNAs (2 μg) were reverse-transcribed to cDNA. Real-time PCR with SYBR Green detection was performed using an ABI PRISM 7500 sequence detection system (Applied Biosystems). Glyceraldehyde-3-phosphate dehydrogenase (GAPDH) was used as a control. Primers are listed below: GAD1_F: 5′-GCG GGA GCG GAT CCT AAT A-3′; GAD1_R: 5′-TGG TGC ATC CAT GGG CTA C-3′; CDO1_F: 5′-GAG GGA AAA CCA GTG TGC CTA C-3′; CDO1_R: 5′-CCT GTT CTC TGG TCA AAG GCG T-3′; GAPDH_F: 5′-ATG TTT GTG ATG GGT GTG AA-3′; GAPDH_R: 5′-ATG CCA AAG TTG TCA TGG AT-3; GGT5_F: 5′-TGT TGG GAG AAG ATG GCA GT-3′; GGT5_R: 5′-AAA GGG TGT GTT GAT GGT GC-3′.

### 2.5. RNA Sequencing and Data Analysis

Total RNAs were extracted from the collected tumors with a High-Capacity cDNA Reverse Transcription Kit (Applied Biosystems). The Agilent 2100 Bioanalyzer and the Agilent RNA 6000 Nano kit were used to determine the quality of RNAs, followed by RNA sequencing using Illumina HiSeq 4000, conducted by Genomics, Taiwan (https://www.genomics.com.tw/) (accessed on 21 May 2022). Purified RNA was used for the preparation of the sequencing library using the TruSeq Stranded mRNA Library Prep Kit (Illumina, San Diego, CA, USA) according to the manufacturer’s instructions. Briefly, mRNA was purified from total RNA (1 μg) using oligo (dT)-coupled magnetic beads and fragmented into small pieces under elevated temperature. First-strand cDNA was synthesized using reverse transcriptase and random primers. After the generation of double-stranded cDNA and adenylation on the 3′-end of DNA fragments, adaptors were ligated and purified using the AMPure XP system (Beckman Coulter, Beverly, CA, USA). The quality of the libraries was examined using the Agilent Bioanalyzer 2100 system and a real-time polymerase chain reaction system. The qualified libraries were sequenced on an Illumina NovaSeq 6000 platform with 150-bp paired-end reads generated by Genomics (BioSci & Tech Co., New Taipei City, Taiwan). All experiments were performed in the Genomics Core through the Genomics Core laboratory process (https://www.genomics.com.tw/en) (accessed on 21 May 2022).

**Figure 2:**
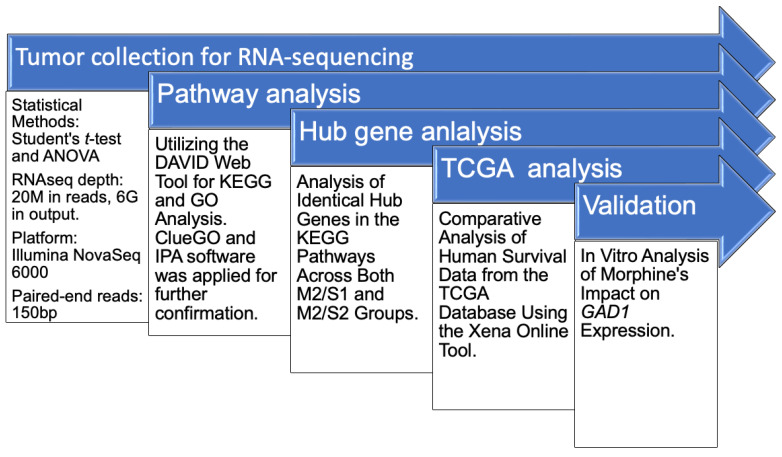
Flowchart of RNA-sequencing data analysis. Total RNA from tumor samples was collected for subsequent RNA sequencing. Approaches and tools for RNA-sequencing data collection and analysis are summarized.

The low-quality bases and sequences from adapters in the raw data were removed using Trimmomatic (version 0.39) [29]. The filtered reads were aligned to reference genomes using Bowtie2 (version 2.3.4.1) [30]. The user-friendly software RSEM (version 1.2.28) was used to quantify transcript abundance [31]. Normalization and identification of differentially expressed genes (DEGs) were carried out using the R package edgeR (v3.24.1). We applied a generalized linear model to estimate the effect sizes and *p*-values of differentially expressed genes between morphine and saline treatments [32,33]. The functional enrichment analysis of Gene Ontology (GO) terms and Kyoto Encyclopedia of Genes and Genomes (KEGG) pathways among gene clusters was performed using an R package called ClusterProfiler (version 3.6.0) [34,35,36] and analyzed using an online bioinformatics tool (Database for Annotation, Visualization, and Integrated Discovery, DAVID; https://david.ncifcrf.gov (accessed on 25 August 2024)) [37]. Other tools, including ClueGo and Ingenuity Pathway Analysis (IPA), were utilized to validate and confirm the significant pathway as a dominant finding [38,39]. The schematic diagram of the data analysis is illustrated in Figure 2.

To determine the relationship between the selected hub genes or pathways and survival, we compared the identified hub genes and pathways using data from the Cancer Genome Atlas (TCGA) database. The UCSC Xena website (https://xenabrowser.net) (accessed on 25 August 2024) is an online database used to determine the correlations among genotypes, phenotypes, and survival in the TCGA breast cancer dataset. We applied hub and transcription genes in UCSC Xena to assess the association of overall survival with gene expression. Patients were divided into high and low gene expression groups for the survival analysis. We determined the number of genes involved in each pathway for the pathway analysis and analyzed the relationship between relapse-free survival and pathway expression [40].

### 2.6. Statistical Analysis

The difference between untreated and different morphine dose-treated samples for MTT and invasion assays was determined using a one-sample Student’s *t*-test. For multiple comparisons, we adjusted the *p*-value using the Bonferroni method. Each morphine-treated sample was normalized to the untreated sample (=1) (Figure 1A,B).

We evaluated the difference in tumor volumes between saline- and morphine-treated groups for 17, 20, and 23 days. We excluded outliers outside the mean ± 1.5 standard deviations to conform to a normal distribution. We used Student’s *t*-test and two-way ANOVA (Figure 1D) for statistical analysis using R (version 4.0.2).

Relative in vitro invasion analysis was performed using Boyden chamber assays, and Student’s *t*-test was used to determine the *p*-value.

## 3. Results

### 3.1. Effect of Morphine on Cell Viability, Invasion, Tumor Growth, and Metastasis

Triple-negative breast cancer is highly metastatic and has a poorer prognosis than other breast cancer subtypes [41]. The EO771 cell line is derived from the C57 BL/6 mouse model of spontaneous breast cancer and is an established model for studying triple-negative breast cancer [42]. To investigate the effect of extended morphine usage on breast cancer cells, EO771 cells were incubated with morphine at concentrations of 0, 1, 10, or 100 μM for 48 h. Our results revealed that up to 100 μM morphine did not affect cell proliferation compared with the mock-treated control. Boyden chamber assays were performed to examine the effect of morphine on the invasion ability of the EO771 cells within 26 h. The EO771 cells treated with ≥1 μM morphine exhibited at least twice the invasion ability of the control group (Figure 1A,B). These results suggest that morphine significantly enhances the invasion ability of EO771 cells but does not affect their proliferation.

While morphine is generally used for pain management, this study focuses on the effect of extended use of morphine on cancer progression. Thus, no additional pain-initiating agent or pain-relief medicine was used to avoid drug–drug interaction. To mimic the effects of extended morphine usage in humans, the mice were intraperitoneally administered 10 mg/kg morphine or the same volume of saline daily for 2 weeks before EO771 cell implantation to the fat pad. The tail-flick test was used to ensure delayed nociception to a heat stimulus for the morphine-treated mice. Figure 1C presents a schematic diagram of the experimental approach, including morphine injection and tumor measurement. Morphine pre-treatment resulted in a significant increase in the tumor volume. Significant differences in tumor volumes were observed between the saline-treated and morphine-treated groups on days 17, 20, and 23 after orthotopic injection of EO771 cells. These findings suggest that morphine administration may have a measurable impact on tumor growth dynamics (Figure 1D). After removing primary tumors on day 38, six saline-treated mice and four morphine-treated mice were followed up based on the health condition of the mice to monitor the potential metastasis till day 68. On the 54th day after tumor cell implantation, lung metastasis developed in three out of the six mice in the saline group and all four mice in the morphine-treated group (Figure 1E and Figure S1). Due to the small sample size, we calculated the statistical power using G*Power (Ver 3.1.9.7) to evaluate whether the sample size was appropriate [43,44]. For the remaining animals—six in the control group and four in the morphine group—the statistical power was 0.96. Although not below the conventional alpha threshold, this trend suggests a potential enhancement of metastatic initiation under morphine treatment. The incidence of lung metastasis was higher in the morphine-treated group compared to the saline-treated group. We further quantified the percentage of metastatic tumor area relative to the total lung area. The mean metastatic area was slightly higher in the morphine group (0.148 ± 0.188%) than in the saline group (0.051 ± 0.023%), but without significant difference (Figure 1F). Together, the results imply that extended morphine usage might promote the initial establishment of metastatic incidence rather than significantly affecting the overall metastatic tumor area within the time frame test.

To determine the mechanism through which morphine affects tumor formation and metastasis, total RNA was isolated from tumors for RNA-sequencing analysis. The DEGs were compared between the morphine-treated and control groups. RNA samples extracted from primary tumors were designated as the control saline 1 (S1) group, and tumors from spontaneous lung metastasis were designated as the saline 2 (S2) group. Tumor RNA samples extracted from the morphine-treated mice on day 38 were morphine 1 (M1). A subset of mice was followed up for metastasis, and RNA was isolated from tumors metastasized to the lung, designated as morphine 2 (M2).

### 3.2. Differential Expression Genes and Functional and Pathway Enrichment Analysis

**Figure 3:**
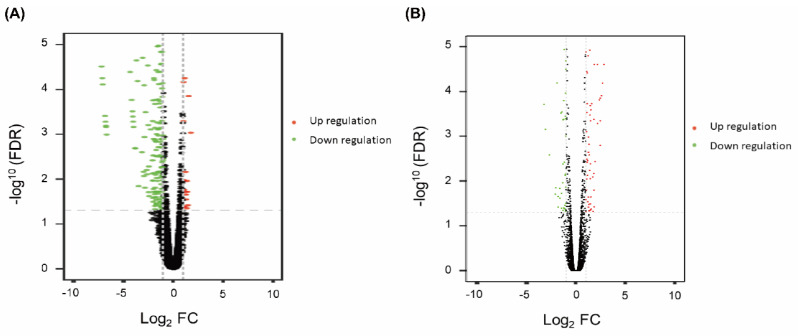
Volcano plots of upregulated and downregulated genes. Upregulated genes are designated in red, whereas downregulated genes are in green. Red spot: dominant upregulated expression (fold-change > 1. FDR < 0.05); green spot: dominant downregulated expression (fold-change < −1, FDR < 0.05); black spot: non-dominant fold-change. (**A**) Comparison of M2 and S1 groups. (**B**) Comparison of M2 and S2 groups.

Unlike previous reports on morphine-mediated tumor growth [45,46], the current study focuses on the effects of extended morphine treatment on TNBC metastasis. We focused on comparing differential expression genes between M2 and S1 (M2/S1 and M2/S2) based on whole-genome RNA-seq data. The volcano plots illustrate the distribution of upregulated and downregulated genes (Figure 3). In the M2/S1 analysis, differentially expressed genes (DEGs) were identified, among which 401 genes were recognized as dominant (false discovery rate [FDR] < 0.05). Of these 401 genes, 32 hub genes were upregulated (Log_2_ FC > 1), and 225 hub genes were downregulated (Log_2_ FC < −1) (Figure 3A). In addition, 12,586 genes were differentially regulated when comparing metastatic M2/S2 tumors. There were 93 dominant upregulation hub genes (FDR < 0.05, Log_2_ FC > 1) and 63 downregulation hub genes (FDR < 0.05, Log_2_ FC < 1), and the volcano plot shows a relatively symmetrical distribution of upregulated and downregulated genes (Figure 3B).

We used the DAVID tool to perform the DEG enrichment analysis, including GO term and KEGG analyses. For RNA-seq analysis of M2/S1, we determined the GO terms and KEGG pathways of the 257 identified genes using DAVID. In the GO analysis, enrichment processes were divided into the biological process (BP), molecular function (MF), and cellular compartment (CC) categories for upregulated genes and downregulated genes (Tables S1 and S2). One upregulation and 10 downregulation pathways of KEGG are listed in Table S3. In the M2/S2 group, KEGG analysis revealed two upregulation pathways of KEGG, and no pathways were identified as significantly downregulated (Table S4). The expression of genes involved in regulating the extracellular matrix was observed to be decreased in this study, consistent with common findings for cancer progression. Genes involved in lipid metabolism, protein/peptide metabolism, and signal transduction in the KEGG analysis were compatible with those identified using GO analysis.

**Figure 4:**
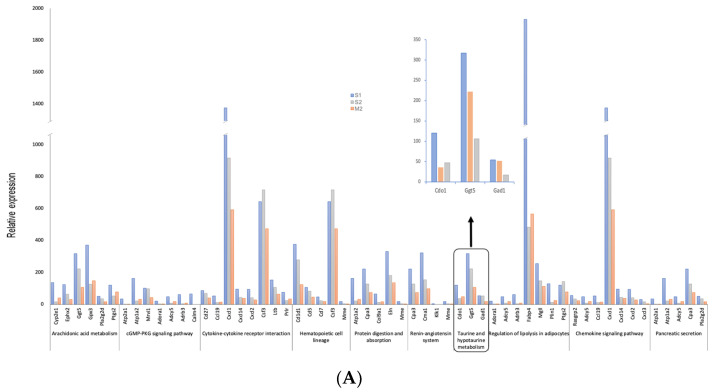

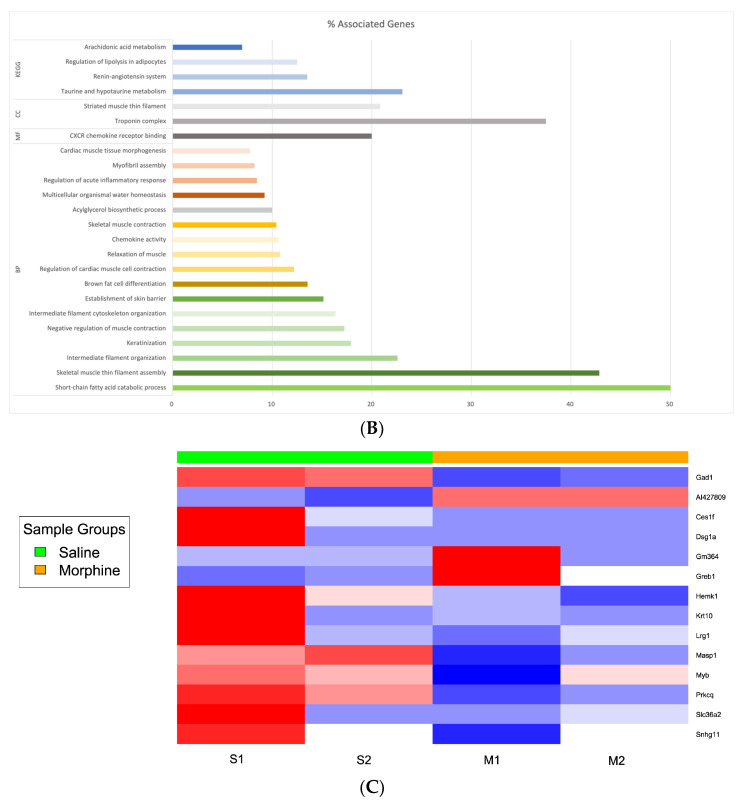
ClueGo analysis to identify significant KEGG pathways and the gene expressions of downregulated KEGG pathways, and heatmap illustration. (**A**) Average expression counts of the S1 (blue), S2 group (gray), and M2 group (orange) are compared. (**B**) The dominant KEGG pathways and the top 20 GO terms are illustrated. Relative expression of 10 downregulated KEGG pathways analyzed using the Cytoscape plugin ClueGo. (**C**) The heatmap shows the differentially regulated genes between the morphine and control groups without metastasis and with metastasis. S1: saline group without metastasis; S2: saline group with metastasis; M1: morphine group without metastasis; M2: morphine group with metastasis. Colors tending toward red indicate upregulation, while colors tending toward blue indicate downregulation.

**Figure 5:**
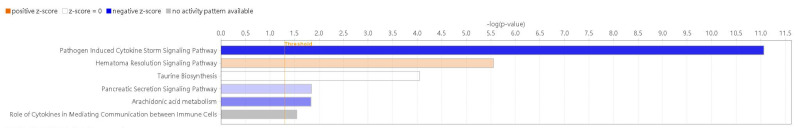
Downregulated pathways revealed by Ingenuity pathway analysis. The illustration shows several downregulated pathways identified using IPA. Taurine biosynthesis and arachidonic acid metabolisms align with the findings from DAVID and ClueGo analyses, reinforcing their importance in the study. The z-score in the figure represents the same regulatory pattern for pathways with identical color gradients. Darker colors indicate more significant regulation. Blue shades correspond to negative z-scores, indicating downregulation, while orange-red shades correspond to positive z-scores, indicating upregulation. White indicates a z-score of 0, meaning that the regulation cannot be determined as either upregulation or downregulation, possibly a result of combined up- and downregulation of genes in the pathway. Gray represents pathways with no activity patterns available.

Based on the M2/S1 downregulated genes, 225 genes were selected for the subsequent analysis (FDR < 0.05). The results of the DAVID analysis revealed 10 downregulated pathways after extended morphine treatment. The relative expression of genes (S1, S2, and M2 counts) within each path is shown in Figure 4A. We then used the Cytoscape (v3.21) plugin ClueGo to integrate the functional pathways identified in the GO term and KEGG analyses of the M2/S1 group. Four dominant KEGG pathways and the top 20 GO terms are illustrated in Figure 4B. To illustrate the differences in gene expression associated with the presence or absence of metastasis between the saline and morphine groups, a heatmap was used as the visual representation (Figure 4C). The downregulated regions were more in number in the M2 group than in the other groups, and the morphine-affected M2 group showed low similarity compared to the control groups. The GO term analysis indicated the dominant downregulation of pathways by morphine treatment. The KEGG analysis revealed dominant downregulation of arachidonic acid, lipolysis, the renin-angiotensin system, and taurine/hypotaurine metabolism. The variation in the KEGG pathways does not elucidate the mechanisms underlying tumor metastasis. In the IPA results, taurine biosynthesis and arachidonic acid metabolism were consistent with the findings from both the DAVID and ClueGo analyses (Figure 5). A previous study reported that the arachidonic acid level may not exert a causal effect on cancer growth. Other metabolites involved in the arachidonic acid pathway, such as 20-hydroxy-eicosatetraenoic acid (*HETE*), may promote tumor growth and invasion [47,48]. In our study, we noted the downregulation of genes involved in arachidonate metabolism, including *PTGS2*, epoxide hydrolase 2, and phospholipase A2. Extended morphine usage is related to the inhibition of substance P [49], and substance P induces the arachidonate cascade by stimulating 12-HETE [50]. Therefore, the definitive correlation between tumor metastasis and arachidonate metabolism should be investigated in future studies.

In comparing the dominant hub genes of the M2/S1 and M2/S2 groups, 16 hub genes were uniformly upregulated, and 29 were uniformly downregulated. Of the 45 hub genes analyzed, only four were exclusive to the M2/S1 or M2/S2 KEGG pathways. One hub gene (*Hspa1b*) associated with upregulation and three with downregulation (*Gad1*, *Mrvi1*, and *Cd1d1*) were involved in the M2/S1 KEGG pathways, while no identical hub genes were found in the M2/S2 pathways (Table S5). Hspa1b, which encodes a heat-shock protein, is involved in the upregulated KEGG pathway associated with legionellosis. Upregulation of this pathway has been reported as a factor for poor outcomes [51]. *GAD1* encodes the key enzyme for taurine synthesis and participates in the KEGG pathway of taurine/hypotaurine metabolism. The *Mrvi1* protein is implicated in the cGMP-PKG signaling pathway and may be related to poor survival [52]. *CD1d1* and *CD1d2* co-encode the CD1d protein, which is critical for developing natural killer T cells, and downregulation of CD1d promotes breast cancer metastasis [53,54]. Notably, one novel discovery is that morphine influences *GAD1* expression; GAD1 is a crucial protein in taurine/hypotaurine metabolism and is associated with the three enzymes responsible for taurine biosynthesis.

**Figure 6:**
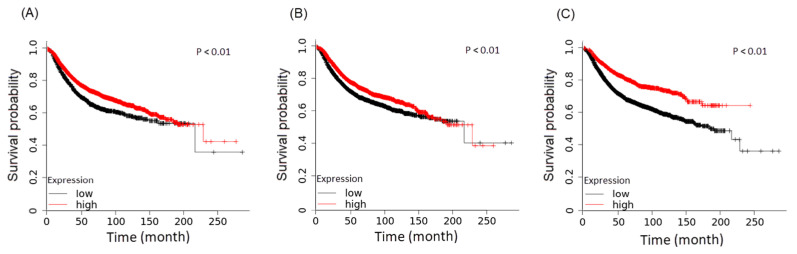
Kaplan–Meier illustration of Kyoto Encyclopedia of Genes and Genomes dominant pathway. Association of high/low expression of genes with survival probability using TCGA Breast Cancer (BRCA) datasets. Kaplan–Meier plot of (**A**) arachidonic acid, (**B**) renin-angiotensin, and (**C**) taurine and hypotaurine metabolism. Red line: effect of higher expression of each pathway on survival probability; black line: effect of lower expression of each pathway on survival probability; *p* < 0.05 indicates a significant difference.

The findings of ClueGo analysis revealed that downregulated KEGG pathways—arachidonic acid metabolism, regulation of lipolysis in adipocytes, the renin–angiotensin system, and taurine and hypotaurine metabolism—correlated with a decreased survival rate (Figure 6). The findings revealed the involvement of taurine and hypotaurine metabolic processes in morphine regulation. To determine the role of taurine synthesis genes in breast cancer patients, we analyzed the candidate genes using TCGA Breast Cancer (BRCA) datasets (*n* = 1097). The BRCA dataset is a cohort database and includes information on variations in gene copy number (*n* = 1080), DNA methylation (*n* = 345), and phenotypes (*n* = 1236). In the TCGA database, higher gene expression levels related to taurine and hypotaurine metabolism, renin–angiotensin signaling, and arachidonic acid metabolism were associated with improved survival outcomes. However, the regulation of lipolysis in adipocytes did not show a significant difference (Figure 6 and Figure S2). Based on these findings, we hypothesized that taurine deficiency may contribute to TNBC progression and increased lung metastasis.

### 3.3. Effect of Taurine on Morphine-Mediated Cell Invasion

**Figure 7:**
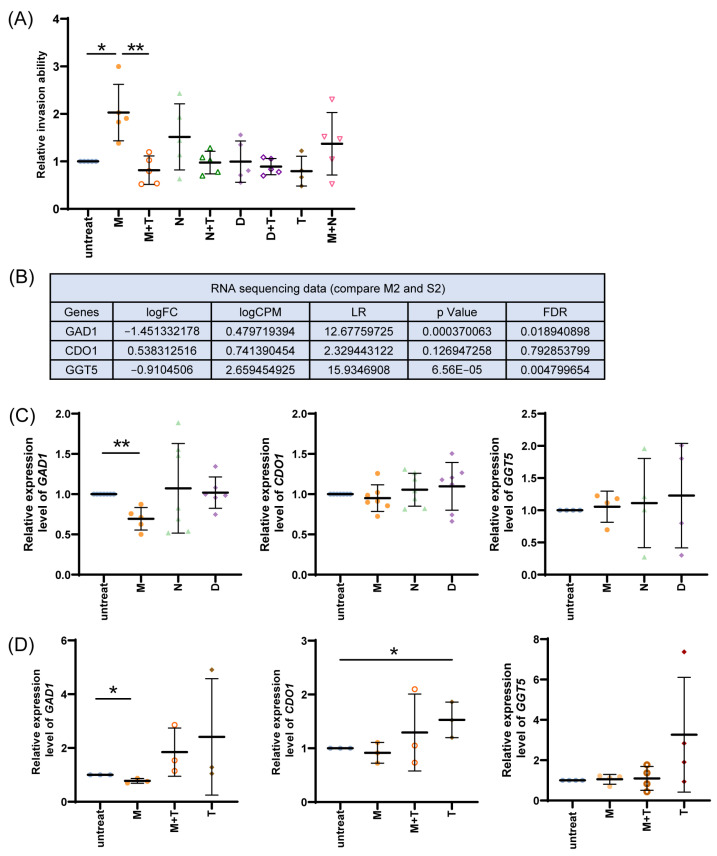
Effect of morphine, naloxone, and droperidol treatment on EO771 cell invasion with or without taurine and on expressions of *GAD1*, *CDO1*, and *GGT5* genes. (**A**) EO771 cells were treated with 10 μM morphine, 1 μM naloxone (opioid receptor antagonist), or 10 μM droperidol (dopamine receptor antagonist) with or without 20 mM taurine every day for 4 days. Boyden chamber assays were then performed to determine relative invasion ability for 26 h. (**B**) RNA sequencing was performed on tumors derived from salinetreated or morphine-treated mice. S2: saline-treated mice with lung metastasis; M2: morphine-treated mice with lung metastasis. (**C**) EO771 cells were treated with 10 μM morphine, 1 μM naloxone, or 10 μM droperidol every day for 4 days. RNAs of EO771 cells were collected. Relative expression levels of *GAD1*, *CDO1*, and *GGT5* genes were determined using qPCR, normalized to *GAPDH* level and no treatment group. (**D**) EO771 cells were treated with 10 μM morphine with or without 20 mM taurine and 20 mM taurine alone every day for 4 days. RNAs of EO771 cells were collected. Relative expression levels of *GAD1*, *CDO1*, and *GGT5* genes were determined using qPCR, normalized to *GAPDH* level and no treatment group. M: morphine; N: naloxone; D: droperidol; T: taurine. (* indicates a significant difference of *p* < 0.05 between groups; ** indicates a significant difference of *p* < 0.01 between groups, determined using Student’s *t*-test and Mann–Whitney U test.) Data were collected from at least three independent experiments. We performed Dixon’s Q test, and the results indicated that none of the data points met the criteria for exclusion.

If the effect of reduced taurine and increased invasion is morphine-opioid receptor-specific action, then naloxone, an opioid receptor antagonist, is not expected to exert a similar effect. Naloxone is a competitive opioid receptor antagonist with a high affinity for the mu-opioid receptor (MOR) and a lower affinity for the kappa-opioid receptor (KOR) and delta-opioid receptor (DOR). It is commonly used as a reversal agent to treat opioid overdose and toxicity, such as respiratory depression and coma [55]. Moreover, morphine has previously been reported to trigger physiological action through dopamine receptor [56]. Droperidol is an antagonist of the dopamine D2 receptor, effectively blocking dopaminergic signaling. It is commonly used to treat the symptoms of nausea and vomiting [57]. Additionally, droperidol acts as an antagonist of the alpha-adrenergic receptor, which can cause hypotension, potentially leading to dangerous conditions in patients. If this is true, droperidol, a dopamine receptor antagonist, would reduce invasion ability. To this end, the EO771 cells were treated with morphine (M), morphine + taurine (M + T), naloxone (N), naloxone + taurine (N + T), droperidol (D), droperidol + taurine (D + T), taurine alone (T), and morphine + naloxone (M + N) over a 4-day period to assess their individual and combined impacts on cellular invasion. As shown in Figure 7A, there was a significant increase in cell invasion with morphine treatment compared to the controls. This effect was mitigated when taurine was added (M + T), highlighting the potential of taurine to counteract morphine-induced cellular invasion. No significant differences were observed among the D, N, untreated, and T groups or between the treatments with and without taurine (D vs. D + T and N vs. N + T). Treatment with N, D, and M + N did not significantly increase invasion either. Extended treatment with morphine similarly increased cell invasion (Figure S3). This result indicates that MOR may play a role, but may not be the only mediator of morphine-induced invasion, consistent with prior reports that morphine promotes cancer cell aggressiveness via additional or alternative signaling pathways [15,21,58]. Nonetheless, longer in vitro treatment inevitably involved multiple cell trypsinization and re-plating; the confounding effect of the cell cycle may have a role on cell behavior. These results reveal a distinct mechanism by which morphine-opioid receptor-specific action influences the pro-invasive behavior of cancer cells, which is reversed by taurine.

Based on our RNA-sequencing results, one key enzyme of taurine biosynthesis, GAD1, was significantly reduced in tumors derived from morphine-treated mice with lung metastasis (M2) compared to those from saline-treated mice with lung metastasis (S2). Another enzyme, cysteine dioxygenase 1 (CDO1), did not differ between these two groups. Although GGT5 downregulation was statistically significant, it was not considered biologically meaningful (log FC > −1) (Figure 7B). To determine whether morphine treatment modulates the expression of these enzymes, EO771 cells were treated with morphine, droperidol, or naloxone for 4 days. Only morphine treatment reduced the expression of *GAD1* by 20%. Consistent with our RNA-sequencing results, *CDO1* expression was not affected by morphine, droperidol, or naloxone (Figure 7C). Compared to the in vivo tumor data, morphine treatment showed a more profound reduction (50%) in *GAD1* (Figure 7B,C). To further investigate whether taurine modulates enzyme expression changes induced by morphine, EO771 cells were treated daily for 4 days with 10 μM morphine alone, 10 μM morphine combined with 20 mM taurine, or 20 mM taurine alone. As shown in Figure 7D, the expression levels of *GAD1* slightly increased with morphine and taurine co-treatment compared to morphine treatment, which corresponds to the rescued invasive phenotype with morphine plus taurine treatment. And the expression levels of *CDO1* increased with taurine treatment. GGT5 acts in downstream taurine catabolism and detoxification processes; it remains uncertain whether exogenous taurine alone would lead to a straightforward upregulation of GGT5. These findings support that taurine may partially rescue morphine-induced *GAD1* downregulation, which correlates with reduced cell invasion.

The detailed workflow for RNA-sequencing data collection and analysis, including the key approaches and bioinformatics tools employed for processing, alignment, differential expression analysis, and pathway enrichment assessments, is illustrated in Figure 2. We focused on the comparison of differential expression genes between M2 and S1 (M2/S1 and M2/S2) based on whole-genome RNA-seq data, followed by functional annotation analysis and experimental validation to further explore the biological significance of these differentially expressed genes (Figure 2). Together, these analyses highlight the novel role of extended morphine treatment in modulating the invasive behavior of TNBC cells through *GAD1* gene expression and demonstrate the potential of taurine as a therapeutic adjuvant to mitigate the invasive effects associated with morphine treatment.

## 4. Discussion

Whether morphine promotes or inhibits tumor formation remains controversial [18], possibly due to the diversity of tumor types, stages, and durations of morphine usage. As morphine has been implicated in immune modulation [59,60], its role in cancer metastasis is not clear. To our knowledge, no study has investigated the interplay between extended morphine exposure and taurine metabolism in TNBC, especially in an immunocompetent animal model.

The findings from this study showed that extended morphine treatment increased the invasive ability of TNBC, which correlated to reduced expression of *GAD1*, encoding the key enzyme for taurine biosynthesis. This downregulation of *GAD1* may represent a novel mechanistic link between long-term morphine usage and metastatic potential. *GAD1* is traditionally known for its role in γ-aminobutyric acid (GABA) biosynthesis in neurons. However, in certain contexts, it participates in the metabolic pathway of taurine synthesis. Taurine is an abundant free amino acid and is involved in anti-inflammation and tumor suppression [16]. In addition, taurine has been reported to enhance the efficacy of chemotherapy and induce cancer cell apoptosis [61]. The mechanism of the anti-tumor effect of taurine may be through suppressing extracellular signal-regulated kinase/ribosomal S6 kinase signaling [16,61,62]. Thus, decreased taurine levels are likely associated with poor prognosis. A recent report showed that diminished taurine levels can lead to exhaustion of CD8+ T cells, whereas supplementation with taurine may enhance therapy [63]. Taurine regulates TME through multiple mechanisms. Taurine normalizes pH in the microenvironment by reducing lactate accumulation, a hallmark of the Warburg effect [17]. Taurine also limits oxidative stress via modulation of glutathione, and other antioxidant defenses may further stabilize TME, preventing the evolution of invasive phenotype [64]. Consequently, diminished *GAD1* expression could impair taurine production locally, facilitating more acidic or immunosuppressive TME, which promotes cancer cell invasion and metastasis. Our findings suggest that morphine-induced *GAD1* downregulation and the subsequent reduction of taurine result in a metastatic phenotype in TNBC.

While previous research has focused on opioid roles in chronic or acute pain management, our findings underscore the importance of chronic morphine exposure—a scenario that many cancer patients experience before or during a breast cancer timeline. Our findings suggest that taurine supplementation may serve as a potential adjuvant therapy to inhibit morphine-induced TNBC metastasis. To establish a clinical protocol, future studies should assess the efficacy and safety of taurine supplementation in TNBC patients with prior morphine treatment. A randomized controlled clinical trial would provide critical evidence regarding the appropriate dosage and therapeutic effects of taurine, while real-world data could further explore whether taurine supplementation directly impacts metastasis formation or improves overall survival when combined with standard chemotherapy or immunotherapy.

Although there is no significant change between M and M + N, prior literature has shown that naloxone or genetic knockout of MOR can reverse morphine-induced tumorigenic actions in breast cancer and other tumor types [15,21]. Nonetheless, based on the existing literature, morphine’s tumor-promoting actions—including enhanced invasiveness—may not be exclusively mediated by classical opioid receptors. For instance, some studies have documented that even in the presence of naloxone, morphine can still promote cancer cell invasion, implying additional or alternative mechanisms [58]. Previous studies simply demonstrating that naloxone does or does not block morphine-induced invasion do not necessarily confirm the exact receptor mechanism, especially with respect to how morphine downregulates *GAD1* in our model. On the other hand, *GGT5* expression was not decreased by morphine treatment, suggesting that *GGT5* may not be involved in the pro-invasive mechanisms of morphine, which appear to be specifically associated with early-stage taurine biosynthesis. We also evaluated how taurine supplementation alone (T) or in combination with morphine (M + T) affects the expression of *GAD1*, *CDO1*, and *GGT5*. Consistent with our previous findings, taurine combined with morphine restored *GAD1* expression. These results reinforce our conclusion that exogenous taurine supplementation can mitigate morphine-induced reduction in *GAD1* expression. Future research using additional opioid antagonists or MOR-knockout systems could further clarify the precise contribution of μ-opioid receptor signaling to *GAD1* downregulation and metastatic progression in TNBC.

**Figure 8:**
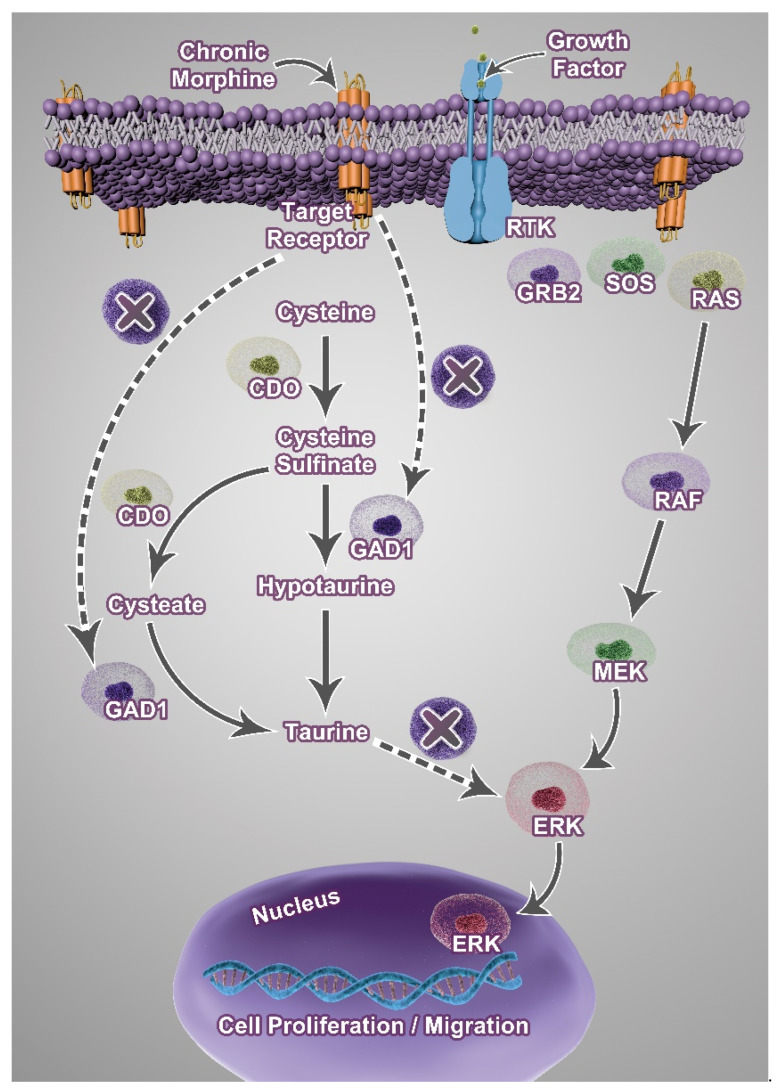
Illustration of morphine-mediated suppression of taurine levels. Morphine acts through candidate target receptors (μ-opioid receptor and/or dopamine receptor) and reduces the expression of *GAD1*, whose activity is responsible for biosynthesis of taurine. Taurine attenuates tumor progression by inhibiting the ERK pathway. *GAD1*: glutamate decarboxylase 1; *CDO1*: cysteine dioxygenase; RTK: receptor of tyrosine kinase.

In line with our study, we showed that extended treatment with morphine indicates a trend toward increased lung metastasis for TNBC. Silencing *GAD1* genes has been reported as a poor prognosis indicator for patients with renal cell carcinoma [65]. Consistent with these findings, our results indicated that extended morphine usage led to decreased *GAD1* expression in TNBC, which may be the key mechanism underlying the reduction in taurine levels (Figure 8). Through human triple-negative breast cancer dataset analysis, *GAD1* expression is stage-dependent, with an initial increase and then decrease in stage IV, comparing metastatic versus non-metastatic cases (Figure S4). Because extended morphine exposure may specifically affect the tumor microenvironment rather than exerting a systemic effect on taurine levels, monitoring local taurine fluctuations in TNBC tissue might be crucial for understanding clinical outcomes.

It is well known that morphine’s immunomodulatory effects may be more apparent in immunocompetent hosts, potentially enhancing metastasis. Previous studies have reported morphine’s effects on tumor growth and metastasis, but the results vary depending on the experimental model and conditions. For example, morphine has been reported to suppress angiogenesis in certain animal models [66], whereas angiogenesis effects have been observed in immunodeficient models [21]. By contrast, our study focuses on an immunocompetent model with extended morphine administration before and after cancer cell implantation, which more closely simulates clinical morphine usage. Our finding of enhanced invasive ability in TNBC models is compatible with studies reporting a pro-metastatic mechanism [20]. Nevertheless, additional research is necessary to reconcile conflicting reports, such as morphine’s suppression of metastasis. The discrepant studies highlight that the extracellular microenvironment, tumor subtype, and immune status are important factors determining morphine’s effects on tumor progression.

Our report reveals that extended exposure of TNBC patients to morphine is a risk factor. Extended morphine exposure appears to affect *GAD1* expression and reduce taurine, specifically within the cancer tissue microenvironment, rather than exert systemic effects. This localized reduction complicates the assessment of taurine variability in TNBC patients. We suggest exploring taurine as a therapeutic supplement in cancer patients requiring long-term opioid analgesics. By bridging pain management and metabolic reprogramming in TNBC, we wish to conduct further investigations that integrate opioid pharmacology, taurine metabolism, and tumor immunology to improve patient prognosis.

Our study has several limitations. First, the sample size in our animal study was relatively small. Although we observed a trend toward increased metastasis in morphine-treated mice, the difference was not statistically significant due to the limited number of animals, which prevents us from making definitive/strong conclusions. Second, we did not measure taurine levels in tumor tissue or plasma, making it difficult to confirm the extent of taurine depletion. The standard taurine level in clinical assessments remains unclear, and determining the optimal dosage for taurine supplementation is also challenging. Third, we did not investigate the effects of taurine supplementation in vivo. Given the complexity of the tumor microenvironment, taurine supplementation may not fully counteract the effects induced by morphine. Moreover, clinical trials are necessary to determine a safe and effective taurine dosage for patients undergoing extended morphine treatment, even though morphine is considered safe in general.

## 5. Conclusions

This study reveals one of the mechanisms underlying morphine-mediated metastasis of triple-negative breast cancer, specifically the reduction of the taurine biosynthesis enzyme GAD1. These findings suggest that taurine supplementation could be explored as a potential adjuvant therapy for TNBC patients undergoing extended morphine treatment, though further clinical examination is required.

## Data Availability

The datasets generated and/or analyzed during the current study are available in the Figshare repository, https://doi.org/10.6084/m9.figshare.22644256.v3, accessed on 25 August 2024.

## Supplementary Materials

The following supporting information can be downloaded at https://www.mdpi.com/article/10.3390/cancers17071086/s1: Figure S1: Metastasis of fat-pad inoculated EO771 cells into the lung tissue; Figure S2: Illustration of Kaplan–Meier lipolysis in adipocytes based on the dominant pathway from the Kyoto Encyclopedia of Genes and Genomes; Figure S3: Effect of morphine, naloxone, and droperidol treatment on EO771 cell invasion with or without taurine; Figure S4: Expression of taurine biosynthesis genes in TCGA database; Table S1: Gene ontology analysis of upregulated differentially expressed genes in the M2/S1 group; Table S2: Gene ontology analysis of downregulated differentially expressed genes in the M2/S1 group; Table S3: Kyoto Encyclopedia of Genes and Genomes analysis of the M2/S1 group; Table S4: Gene ontology (GO) and Kyoto Encyclopedia of Genes and Genomes (KEGG) analysis of upregulated differentially expressed genes in the M2/S2 group; Table S5: Upregulation and downregulation of identical gene in both M2/S1 and M2/S2.

## Abbreviations

GPCR	G-protein-coupled receptor
MOR	μ-opioid receptor
NK	natural killer
FBS	fetal bovine serum
MTT	3-(4,5 dimethylthiazol-2-tl)-2,3-diphenyltetrazolium bromide
BSA	Bovine serum albumin
NTHU	National Tsing Hua University
GAPDH	Glyceraldehyde-3-phosphate dehydrogenase
FDR	False Discovery Rate
KEGG	Kyoto Encyclopedia of Genes and Genomes
GO	Gene Ontology
DEGs	Differentially expressed genes
DAVID	Database for Annotation, Visualization, and Integrated Discovery
TCGA	Cancer Genome Atlas
GAD1	Glutamate decarboxylase 1
BP	Biological process
MF	Molecular function
CC	Cellular compartment
CDO1	Cysteine dioxygenase 1
CD1d1	Cluster of Differentiation 1-d1
Hspa1b	Heat Shock Protein a1b
Mrvi1	Murine Retrovirus Integration site 1
