# Supplementary material for: Reduced Taurine Synthesis Underlies Morphine-Promoted Lung Metastasis of Triple-Negative Breast Cancer
Source: Cancers (Basel). 2025 Mar 24;17(7):1086. doi: 10.3390/cancers17071086 (PMC11988058; doi:10.3390/cancers17071086)
Supplement: Supplementary file 1 [file cancers-17-01086-s001.zip › Figures.pdf]

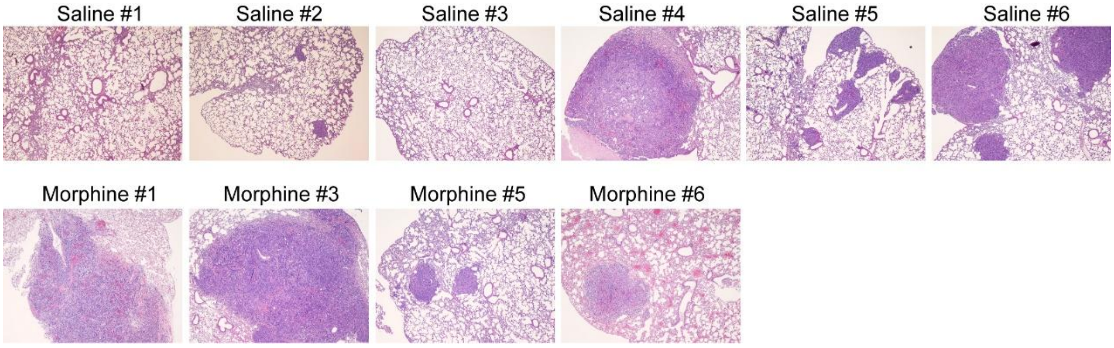

Figure S1. Metastasis of fat-pad inoculated EO771 cells into the lung tissue. The relative area of tumors in the lung for saline-treated and morphine-treated mice. The H.E.-stained tissue section image shows that in the saline group, 3 out of 6 mice exhibited lung metastasis, whereas in the morphine group, all 4 mice exhibited lung metastasis.

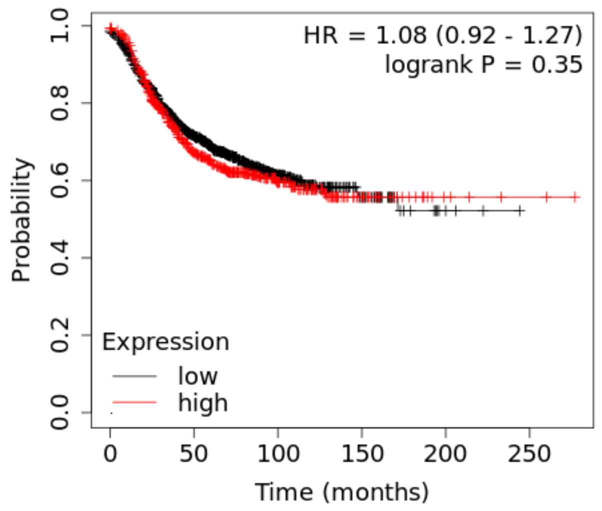

Figure S2. Kaplan–Meier lipolysis in adipocyte illustration of Kyoto Encyclopedia of Genes and Genomes dominant pathway.

Association of high/low expression of genes with survival probability by using TCGA Breast Cancer (BRCA) datasets. Kaplan–Meier plot of regulation of lipolysis in adipocyte. It shows no significant difference in the survival of expression.

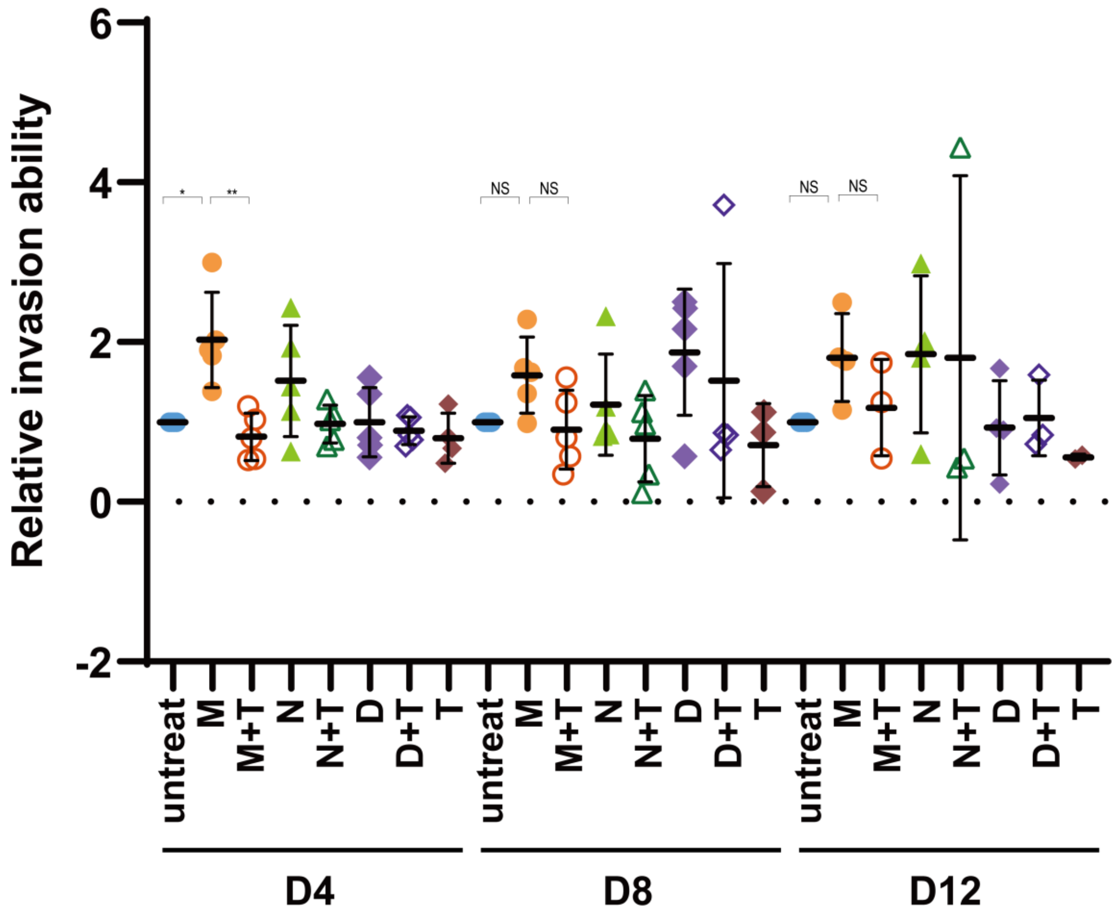

Figure S3. Effect of morphine, naloxone and droperidol treatment on EO771 cell invasion with or without taurine

A significant increase in cell invasion was observed with morphine treatment (M) compared to controls, particularly notable at D4, D8, and D12. This effect was mitigated when taurine was added (M+T), demonstrating the potential of taurine to counteract morphine-induced cellular invasion. No significant differences were found among the D, N, untreated, and T groups, nor between the treatments with and without taurine (D vs. D+T and N vs. N+T). Furthermore, neither N nor D treatments significantly increased invasion ability, highlighting the specific impact of morphine on cellular invasion over time. (* indicates a significant difference of $P < 0.05$ between groups; ** indicates a significant difference of $P < 0.01$ between groups, NS indicates no significant difference, determined using the Students' *t*-test.)

M: morphine; N: naloxone; D: droperidol; T: taurine.

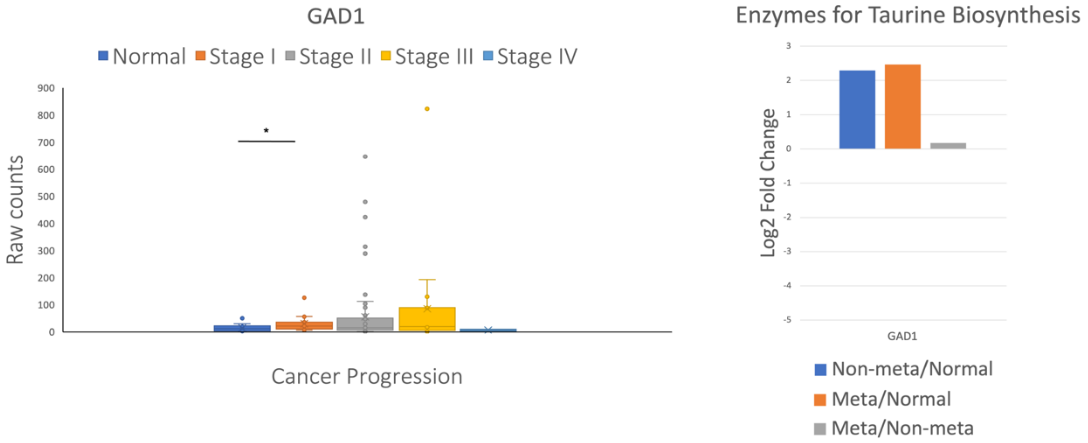

Figure S4. Expression of taurine biosynthesis genes in TCGA database

Fold change of gen *GAD1* expression. Raw counts of *GAD1* was compared in groups of normal tissue and stage I-IV. Relative non-meta/normal, meta/normal, and meta/non-meta level of *GAD1* expression was shown.
