# Supplementary material for: Reduced Taurine Synthesis Underlies Morphine-Promoted Lung Metastasis of Triple-Negative Breast Cancer
Source: Cancers (Basel). 2025 Mar 24;17(7):1086. doi: 10.3390/cancers17071086 (PMC11988058; doi:10.3390/cancers17071086)
Supplement: Supplementary file 1 [file cancers-17-01086-s001.zip › Tables.pdf]

Table S1. Gene ontology analysis of upregulated differentially expressed genes in M2/S1 group.

**Category**	**Term**	**Count**	**Genes**	***FDR**
Biological process	Cytolysis	5	*GZMC*, *GZME*, *GZMD*, *GZMG*, *GZMF*	<0.01
	Protein processing	5	*GZMC*, *GZME*, *GZMD*, *GZMG*, *GZMF*	<0.01
	Immune response	5	*GZMC*, *GZME*, *GZMD*, *GZMG*, *GZMF*	<0.01
	Proteolysis	5	*GZMC*, *GZME*, *GZMD*, *GZMG*, *GZMF*	<0.01
Cellular compartment	Intracellular membrane-bounded organelle	6	*GZMC*, *OLR1*, *GZME*, *GZMD*, *GZMG*, *GZMF*	<0.01
Molecular function	Serine-type peptidase activity	5	*GZMC*, *GZME*, *GZMD*, *GZMG*, *GZMF*	<0.01
	Serine-type endopeptidase activity	5	*GZMC*, *GZME*, *GZMD*, *GZMG*, *GZMF*	<0.01
	Peptidase activity	5	*GZMC*, *GZME*, *GZMD*, *GZMG*, *GZMF*	<0.01
	Hydrolase activity	6	*GZMC*, *GZME*, *GZMD*, *GZMG*, *HDAC9*, *GZMF*	<0.01

*FDR(False Discover Rate) is the probability of the number of genes out of the total gene number in the GO term list. The genes in each GO term are compared with the background distribution of annotation. A lower FDR represents the association of significant GO terms with the group of genes. FDR < 0.05 indicates a significant association.

Table S2. Gene ontology analysis of downregulated differentially expressed genes in M2/S1 group.

Category	Term	Count	Gene	FDR
Biologic process	Immune response	15	H2-EB2, CSF3, CMA1, MCPT4, SUSD2, CXCL1, CXCL3, CXCL14, CXCL2, MYLPF, ENPP2, CD27, LTB, CCL19, TNFSFM13	<0.01
	Inflammatory response	15	GGT5, TPSB2, RARRES2, EPHX2, CXCL1, CXCL3, PTGS2, CXCL2, KRT16, CD27, PRKCQ, CHIL3, FFAR2, CCL19, S100A9	<0.01
	Lipid metabolic process	14	PLA2G2D, DGAT2, EPHX2, PTGS2, CES1D, CYP11A1, PNPLA3, ENPP2, PLCE1, THRSP, PLIN1, PCK1, SLC27A3, MGLL	<0.01
	Cell adhesion	14	AOC3, MYBPC2, LAMA3, PCDH15, DSG1A, CELSR2, SELL, PERP, SSPO, PKP1, CD226, SVEP1, CDH15, DSC3	<0.01
	Proteolysis	14	CPA3, GGT5, CFD, TPSB2, KLK1, MME, CMA1, MCPT4, HP, TMPRSS2, ASPRV1, KLK7, MASP1, LTF	0.01
	Immune system process	13	CFD, IFITM1, HP, MARCO, CD1D1, KRT16, CD7, LCN2, PRKCQ, FFAR2, MASP1, S100A9, LTF	<0.01
	Keratinization	12	TGM1, SPRR2A2, KRT17, KRT16, SPRR2A1, SFN, PPL, KRT6B, SPRR1B, LOR, HRNR, KRT6A	<0.01
	Metabolic process	11	CHIT1, ISOC2B, EPHX2, PNPLA3, ENPP2, AMY1, CHIL3, SLC27A3, ALDH1A7, UGT1A6B, ENGASE	0.03
	Keratinocyte differentiation	10	DSP, TGM1, SPRR2A2, KRT16, SPRR2A1, TRP63, SFN, KRT10, PTGS2, SPRR1B	<0.01
	Brown fat cell differentiation	8	LRG1, FABP4, ADRB3, RARRES2, ADIPOQ, SLC2A4, PTGS2, ADIG	<0.01

	Lipid catabolic process	8	PLA2G2D, CES1D, ADORA1, PNPLA3, ENPP2, PLCE1, PLIN1, MGLL	<0.01
	Response to lipopolysaccharide	8	CITED1, CD27, FMO1, CXCL1, PCK1, PTGS2, CXCL3, CXCL2	<0.01
	In utero embryonic development	8	ANGPT1, RARRES2, AMD1, ENO1B, HBA-A2, FGFR2, DSC3, TTN	0.04
	Morphogenesis of an epithelium	7	CITED1, KRT17, KRT16, PCDH15, KRT6B, SERPINB5, KRT6A	<0.01
	Cell chemotaxis	7	SAA3, ENPP2, PRKCQ, CXCL1, CCL19, CXCL14, CXCL2	<0.01
	Response to ethanol	7	CAR3, CSF3, ADIPOQ, CD27, SLC2A4, CYP2E1, CDO1	<0.01
	Cardiac muscle contraction	6	CSRP3, MYL1, TCAP, TNNI2, SCN5A, TTN	<0.01
	Cellular response to tumor necrosis factor	6	FABP4, GPD1, LCN2, SLC2A4, CCL19, PCK1	<0.01
	Glucose homeostasis	6	ADIPOQ, PDK4, FFAR2, SLC2A4, MUP11, PCK1	0.02
	Homophilic cell adhesion via plasma membrane adhesion molecules	6	PCDH15, CD226, CDH15, DSG1A, CELSR2, DSC3	<0.01
	Intermediate filament organization	5	DSP, KRT17, KRT10, KRT6B, KRT6A	<0.01
	Regulation of muscle contraction	5	TNNC2, TNNT3, TNNI2, ATP2A1, ATP1A2	<0.01
	Positive regulation of neutrophil chemotaxis	5	SELL, CXCL1, CCL19, CXCL3, CXCL2	<0.01
	Muscle contraction	5	MYH1, MYBPC2, MYL1, MYH4, TTN	<0.01

	Peptide cross-linking	5	DSP, TGM1, SPRR2A2, SPRR2A1, SPRR1B	<0.01
	Epidermis development	5	KRTDAP, SPRR2A2, SPRR2A1, TRP63, KRT10	<0.01
	Epithelial cell differentiation	5	CES1D, KRT14, TRP63, KRT10, FGFR2	<0.01
	Neutrophil chemotaxis	5	CXCL1, CCL19, CXCL3, CXCL2, S100A9	<0.01
	Cellular response to interleukin-1	5	SAA3, LCN2, CCL19, PCK1, CXCL2	0.01
	Response to estradiol	5	SPRR2A2, SPRR2A1, PTN, PTGS2, CXCL2	0.03
	Chemotaxis	5	RARRES2, ENPP2, CXCL3, CXCL2, S100A9	0.04
	Notch signaling pathway	5	CFD, KRT19, PERP, HP, TRP63	0.05
	Cardiac muscle tissue morphogenesis	4	ANGPT1, TCAP, XIRP2, TTN	<0.01
	Establishment of skin barrier	4	KRT16, KRT1, TRP63, SFN	<0.01
	Skeletal muscle contraction	4	TNNC2, TNNT3, TCAP, TNNI2	<0.01
	Iron ion homeostasis	4	STEAP4, MFI2, LCN2, LTF	0.01
	Response to activity	4	ADIPOQ, PTN, PCK1, MYH4	0.02
	Cellular response to calcium ion	4	KRT10, SCN5A, RASGRP2, SLC25A23	0.02
	Chemokine-mediated signaling pathway	4	CXCL1, CCL19, CXCL3, CXCL2	0.02
	Skin development	4	DSP, SFN, LTB, ASPRV1	0.03
	Response to glucocorticoid	4	ADIPOQ, CDO1, PTGS2, CXCL2	0.05

	Detection of muscle stretch	3	CSRP3, TCAP, TTN	<0.01
	Skeletal muscle thin filament assembly	3	ACTA1, TCAP, TTN	<0.01
	Regulation of striated muscle contraction	3	TNNT3, ATP2A1, ATP1A2	<0.01
	Skeletal muscle myosin thick filament assembly	3	TCAP, MYH4, TTN	<0.01
	Cardiac muscle hypertrophy	3	CSRP3, TCAP, TTN	<0.01
	Cardiac myofibril assembly	3	CSRP3, TCAP, TTN	<0.01
	Hair cycle	3	KRT16, KRT14, PTGS2	<0.01
	Regulation of epithelial cell proliferation	3	TACSTD2, SERPINB5, FGFR2	0.01
	Positive regulation of NF-kappab import into nucleus	3	PRKCQ, CCL19, PTGS2	0.02
	Triglyceride catabolic process	3	PNPLA3, PLIN1, MGLL	0.02
	Positive regulation of blood pressure	3	EPHX2, ADIPOQ, ADORA1	0.04
	Response to antibiotic	3	AOC3, ADRB3, SLC1A3	0.04
	Sarcomere organization	3	KRT19, TCAP, TTN	0.04
	Squamous basal epithelial stem cell differentiation	2	TRP63, FGFR2	0.02

	involved in prostate gland acinus development			
	Short-chain fatty acid catabolic process	2	CES1D, CES1F	0.02
	Gamma-aminobutyric acid biosynthetic process	2	GAD1, SLC1A3	0.03
	Sarcomerogenesis	2	TCAP, TTN	0.03
	Polysaccharide catabolic process	2	CHIT1, CHIL3	0.03
	Regulation of angiotensin levels in blood	2	CPA3, MCPT4	0.03
	Regulation of epidermal cell division	2	TRP63, SFN	0.03
	Regulation of lipid catabolic process	2	RARRES2, LGALS12	0.04
	Positive regulation of peptide secretion	2	ADORA1, S100A9	0.04
	Negative regulation of striated muscle contraction	2	ATP2A1, ATP1A2	0.04
Molecular function	Structural molecule activity	17	DSP, LAD1, KRT1, KRT79, KRT7, KRT77, KRT5, KRT10, KRT19, SPRR2A2, KRT17, KRT16, KRT15, SPRR2A1, KRT14, KRT6B, KRT6A	<0.01
	Hydrolase activity	27	CFD, TPSB2, KLK1, MCPT4, TMPRSS2, ATP2A1, ATP1A2, KLK7, ENPP2, PLCE1, CPA3, GGT5, PLA2G2D, MME, CMA1, EPHX2, ASPRV1,	0.02

			CHIT1, CES1D, CES1F, PNPLA3, AMY1, CHIL3, MASP1, MGLL, ENGASE, LTF	
	Calcium ion binding	24	AOC3, PLA2G2D, TNNC2, PCDH15, ATP2A1, DOC2B, RASGRP2, DSG1A, CELSR2, HRNR, TTN, MYLPF, MYL1, ENPP2, ANXA8, AMY1, SVEP1, SLIT3, CDH15, MASP1, CALM4, SLC25A23, S100A9, DSC3	<0.01
	Protein homodimerization activity	20	AOC3, ABCD2, DGAT2, CITED1, EPHX2, ENO1B, ADIPOQ, HP, ATP2A1, PTGS2, GIMAP7, UGT1A6B, ADRB3, GPD1, LCN2, CD226, THRSP, MASP1, FGFR2, MGLL	<0.01
	Peptidase activity	14	CPA3, GGT5, CFD, TPSB2, KLK1, MME, CMA1, MCPT4, TMPRSS2, ASPRV1, KLK7, KLK6, MASP1, LTF	<0.01
	Serine-type endopeptidase activity	11	CFD, TPSB2, KLK1, CMA1, MCPT4, HP, TMPRSS2, MASP1, KLK7, KLK6, LTF	<0.01
	Serine-type peptidase activity	9	CFD, TPSB2, KLK1, CMA1, MCPT4, TMPRSS2, MASP1, KLK7, LTF	<0.01
	Structural constituent of cytoskeleton	8	MYBPC2, SPRR2A2, KRT16, SPRR2A1, SPTB, SPRR1B, LOR, TTN	<0.01
	Iron ion binding	8	CYP11A1, MFI2, LCN2, CYP4B1, HBA-A2, CYP2E1, CDO1, LTF	<0.01
	Heparin binding	7	TPSB2, PLA2G2D, CCDC80, SLIT3, PTN, FGFR2, LTF	<0.01
	Cytokine activity	7	CSF3, CXCL1, LTB, CXCL3, CXCL14, CXCL2, CRLF1	0.03
	Structural constituent of muscle	5	MYLPF, CSRP3, KRT19, TCAP, TTN	<0.01
	Chemokine activity	5	CXCL1, CCL19, CXCL3, CXCL14, CXCL2	<0.01
	Scavenger receptor activity	5	MARCO, CD5, SUSD2, ENPP2, TMPRSS2	<0.01

	Scaffold protein binding	5	DSP, KRT15, KRT5, SCN5A, ADCY5	<0.01
	Cell adhesion molecule binding	4	DSP, CD1D1, SELL, CD226	0.04
	CXCR chemokine receptor binding	3	CXCL1, CXCL3, CXCL2	<0.01
	Ankyrin binding	3	SCN5A, SPTB, TTN	0.02
	Carboxy-lyase activity	3	GAD1, AMD1, PCK1	0.03
	Peroxidase activity	3	GPX3, HBA-A2, PTGS2	0.05
Cellular component	Membrane	93	TMEM45B, IFITM1, ABCD2, STEAP4, ENO1B, GIMAP3, ATP2A1, KLHDC7A, HBA-A2, DOC2B, SLC2A4, SPINT2, SLC7A10, AQP3, CELSR2, TGM1, CD1D1, B3GALT2, ADORA1, PLCE1, SVEP1, FFAR2, SCN5A, SLC16A2, ADIG, DSP, SLC36A2, DGAT2, MME, GRID1, KRT1, MCEMP1, KRT5, SPTB, PRLR, DSG1A, ADRB3, LY6D, PLIN4, CD226, PRKCQ, CDH15, CYP2E1, LTB, SLC27A3, NAT8L, NPTXR, S100A9, MGLL, DSC3, TMEM45A, SLC45A3, NPY4R, MFI2, TACSTD2, PCDH15, SLC1A3, TMPRSS2, ATP1A2, PTN, PTGS2, RASGRP2, PPL, ADCY5, EXTL1, CYP11A1, PERP, SLIT3, SLC25A23, TNFSFM13, PROM2, SLC38A4, GGT5, AOC3, SUSD2, FMO1, CYP4B1, KRT10, HS3ST6, SYT13, ASPRV1, 5330417C22RIK, MARCO, ADGRD1, SELL, CD5, CD7, PNPLA3, CD27, MRVI1, CD247, FOLR1, FGFR2	0.01
	Extracellular exosome	69	FCGBP, LAD1, STEAP4, ENO1B, HP, HBA-A2, SLC2A4, DMKN, TGM1, ANXA8, KRT6B, KRT6A, DSP, HIST1H2AO, SLC36A2, MME, GPX3, GRID1, KRT1, ADIPOQ, KRT79, KRT7, KRT77, KRT5, DSG1A, MUC5AC,	<0.01

			UGT1A6B, SERPINB5, ACTA1, GPD1, PKP1, AMY1, CDH15, S100A9, LTF, CFD, KLK1, MFI2, LAMA3, TACSTD2, PCDH15, TMPRSS2, RETN, PPL, TTN, SFN, PCK1, PROM2, ANGPT1, EPHX2, SUSD2, RARRES2, KRT10, SERPINA3N, 5330417C22RIK, KRT19, LRG1, FABP4, KRT17, KRT16, CILP, KRT15, KRT14, APOC1, CD7, LCN2, CD27, CALM4, FOLR1	
	Extracellular region	56	LAD1, CSF3, TPSB2, ELN, HP, CXCL1, CXCL3, DMKN, CXCL14, CXCL2, BMPER, ENPP2, SVEP1, KRTDAP, GPX3, ADIPOQ, MUP11, SERPINB5, CHIT1, CCDC80, AMY1, CHIL3, MASP1, S100A9, DSC3, LTF, CFD, RETNLA, MFI2, SAA3, LAMA3, PCDH15, TMPRSS2, PTN, RETN, KLK7, KLK6, APOL6, SFN, SLIT3, PLA2G2D, ANGPT1, CMA1, RARRES2, SERPINA3N, IGSF10, CILP, APOC1, COL9A1, LCN2, SSPO, PTX3, CCDC3, FOLR1, CRLF1, FGFR2	<0.01
	Extracellular space	51	CSF3, TPSB2, ENO1B, HP, CXCL1, CXCL3, DMKN, CXCL14, CXCL2, BMPER, ENPP2, CPA3, GPX3, KRT1, ADIPOQ, MUP11, MUC5AC, SERPINB5, CHIT1, ACTA1, AMY1, MASP1, LTB, NPTXR, S100A9, LTF, CFD, RETNLA, KLK1, MFI2, SAA3, TACSTD2, RETNLG, PTN, RETN, KLK7, KLK6, SLIT3, CCL19, TNFSFM13, AOC3, ANGPT1, KRT10, SERPINA3N, LRG1, CES1D, CILP, CES1F, LCN2, SSPO, PTX3	<0.01
	Endoplasmic reticulum	23	AOC3, SRPX, DGAT2, ADIPOQ, HP, CYP4B1, FMO1, ATP2A1, PTN, PTGS2, UGT1A6B, CIDEC, EXTL1, CES1D, CES1F, APOC1, ADORA1, CHIL3, PLIN1, CCDC3, SCN5A, CYP2E1, NAT8L	0.03

	Integral component of plasma membrane	20	STEAP4, NPY4R, TACSTD2, ATP2A1, ATP1A2, SLC2A4, SYT13, SLC7A10, AQP3, 5330417C22RIK, ADRB3, PERP, ENPP2, CD27, CD226, SLC16A2, FGFR2, SLC38A4, TMEM45A, PROM2	0.04
	Intracellular membrane-bounded organelle	18	AOC3, ABCD2, DGAT2, CMA1, MCPT4, CYP4B1, FMO1, ATP2A1, ATP1A2, SLC2A4, SYT13, PTGS2, UGT1A6B, PLIN4, PKP1, CYP2E1, NAT8L, FGFR2	<0.01
	Cell surface	17	AOC3, MFI2, ADIPOQ, SLC1A3, PTN, SLC2A4, PRLR, CD1D1, SELL, LY6D, CD27, CD226, SCN5A, FOLR1, FGFR2, LTF, PROM2	<0.01
	Intermediate filament	13	DSP, KRT1, KRT79, KRT7, KRT77, KRT5, KRT10, KRT19, KRT17, KRT16, KRT15, KRT14, KRT6A	<0.01
	Synapse	11	MME, GRID1, GAD1, ADORA1, PCDH15, SLC1A3, ATP1A2, SYT13, DOC2B, RASGRP2, MGLL	0.05
	Keratin filament	9	KRT1, KRT14, KRT79, KRT7, KRT77, KRT5, KRT10, KRT6B, KRT6A	<0.01
	Extracellular matrix	9	DSP, TPSB2, CILP, CMA1, RARRES2, KRT1, DSG1A, S100A9, FGFR2	0.01
	Proteinaceous extracellular matrix	9	FCGBP, LAD1, CCDC80, CILP, ELN, LAMA3, COL9A1, SLIT3, PTN	0.02
	Lipid particle	8	DGAT2, CES1D, CES1F, PNPLA3, PLIN4, PLIN1, GIMAP7, CIDEC	<0.01
	Desmosome	6	DSP, PERP, PKP1, PPL, DSG1A, DSC3	<0.01
	Cornified envelope	6	DSP, SPRR2A2, SPRR2A1, SPRR1B, LOR, HRNR	<0.01
	Sarcolemma	6	KRT19, ATP1A2, PRKCQ, SLC2A4, SCN5A, ADCY5	<0.01
	Z disc	6	CSRP3, KRT19, TCAP, XIRP2, SCN5A, TTN	0.01
	Cell periphery	5	KRT19, KRT17, KRT14, ADIPOQ, SLC1A3	<0.01
	Intercalated disc	4	DSP, MYH1, ATP1A2, SCN5A	0.02

	Myosin complex	4	MYLPF, MYH1, MYL1, MYH4	0.02
	Troponin complex	3	TNNC2, TNNT3, TNNI2	<0.01
	Myosin filament	3	MYH1, MYBPC2, MYH4	<0.01
	I band	3	TCAP, ATP2A1, TTN	0.03
	Fibril	2	SLC1A3, MUC5AC	0.04

FDR(False Discover Rate) is the probability of numbers of genes out of total gene in the GO term list. The GO terms shared be the genes in the list are compared to background distribution of annotation. The less FDR present, the significant GO term associates with the group of gene. FDR < 0.05 is defined as significant association.

Table S3. Kyoto Encyclopedia of Genes and Genomes analysis of M2/S1 group.

Pathway name	Count	Genes	FDR
**Up-regulation**<br>Legionellosis	2	*NAIP7, HSPA1B*	0.03
**Down-regulation**<br>Cytokine–cytokine receptor interaction	8	*CSF3, CD27, CXCL1, LTB, CCL19, CXCL14, CXCL2, PRLR*	0.033
Regulation of lipolysis in adipocytes	7	*FABP4, ADRB3, ADORA1, PLIN1, PTGS2, MGLL, ADCY5*	<0.01
cGMP-PKG signaling pathway	7	*ADRB3, ADORA1, ATP2A1, MRVI1, ATP1A2, CALM4, ADCY5*	0.02
Chemokine signaling pathway	7	*CXCL1, CCL19, RASGRP2, CXCL3, CXCL14, CXCL2, ADCY5*	0.04
Arachidonic acid metabolism	6	*GGT5, PLA2G2D, GPX3, EPHX2, CYP2E1, PTGS2*	<0.01
Hematopoietic cell lineage	5	*CSF3, CD1D1, MME, CD5, CD7*	0.02
Protein digestion and absorption	5	*CPA3, MME, ELN, COL9A1, ATP1A2*	0.02
Pancreatic secretion	5	*CPA3, PLA2G2D, ATP2A1, ATP1A2, ADCY5*	0.04
Renin–angiotensin system	4	*CPA3, KLK1, MME, CMA1*	<0.01
Taurine and hypotaurine metabolism	3	*GGT5, GAD1, CDO1*	<0.01

FDR (False Discover Rate) is the probability of the number of genes out of the total gene number in the KEGG list. The genes in the KEGG list are compared with the background distribution of annotation. A lower FDR represents the association of significant KEGG pathways with the group of genes. FDR < 0.05 indicates a significant association

Table S4. Gene ontology (GO) and Kyoto Encyclopedia of Genes and Genomes (KEGG) analysis of upregulated differentially expressed genes in M2/S2 group.

Category	Term/Pathway name	Count	Genes	FDR
**Upregulation of Gene ontology**				
Biological process	cellular oxidant detoxification	4	*Hba-a1, Hbb-b1, Hbb-b2, Hbb-bt*	0.049
	nitric oxide transport	3	*Hba-a1, Hbb-b1, Hbb-b2*	0.049
Cellular compartment	hemoglobin complex	4	*Hba-a1, Hbb-b1, Hbb-b2, Hbb-bt*	< 0.01
	haptoglobin-hemoglobin complex	4	*Hba-a1, Hbb-b1, Hbb-b2, Hbb-bt*	< 0.01
	extracellular space	21	*Ccl12,Cd109,Wfdc1,Wfdc15a,Wfikkn2,Adipoq,Ang,Arg1,Col14a1,Cfd,Gas6,Hba-a1,Hbb-b1,Hbb-b2,Hbb-bt,Lrrc15,Mup10,Mup18,Pf4,Retn,Tgfb2*	< 0.01
	cell periphery	5	*Vangl2,Adipoq,Gap43,Krt19,Lyve1*	0.034
	extracellular region	17	*Ccl12,Cd109,Wfdc1,Wfdc15a,Wfikkn2,Adipoq,Ang,F13a1,Col14a1,Cfd,Qpct,Gas6,Mapt,Pla2r1,Pf4,Retn,Tgfb2*	0.036
	cytolytic granule	3	*Gzmd, Gzme, Gzmg*	0.037
Molecular function	organic acid binding	4	*Hba-a1, Hbb-b1, Hbb-b2, Hbb-bt*	< 0.01
	haptoglobin binding	4	*Hba-a1, Hbb-b1, Hbb-b2, Hbb-bt*	< 0.01
	oxygen transporter activity	4	*Hba-a1, Hbb-b1, Hbb-b2, Hbb-bt*	< 0.01
	hemoglobin beta binding	3	*Hbb-b1, Hbb-b2, Hbb-bt*	< 0.01
	oxygen binding	4	*Hba-a1, Hbb-b1, Hbb-b2, Hbb-bt*	< 0.01
	hemoglobin alpha binding	3	*Hbb-b1, Hbb-b2, Hbb-bt*	0.014
	peroxidase activity	4	*Hba-a1, Hbb-b1, Hbb-b2, Hbb-bt*	0.017

	hemoglobin binding	3	*Hbb-b1, Hbb-b2, Hbb-bt*	0.017
	peptidase inhibitor activity	5	*Cd109,Wfdc1,Wfdc15a,Wfikkn2,Pbp2*	0.034
	serine-type endopeptidase inhibitor activity	5	*Cd109,Wfdc1,Wfdc15a,Wfikkn2,Pbp2*	0.038
**Up-regulation of Kyoto Encyclopedia of Genes and Genomes**				
KEGG	Malaria	6	*Ccl12,Hba-a1,Hbb-b1,Hbb-b2,Hbb-bt,Tgfb2*	< 0.01
	African trypanosomiasis	4	*Hba-a1, Hbb-b1, Hbb-b2, Hbb-bt*	0.036
**Down-regulation of Gene ontology**				
Cellular compartment	extracellular space	15	*Cx3cl1,Cd1d1,Sspo,Angpt1,Bmp5,Cpa6,Hba-a2,Il1rl1,Lcn2,Lum,Nptxr,Prss56,Tslp,Tg,Tnfsfm13*	0.035
	membrane	36	*Cx3cl1,Cd1d1,Deptor,Epha1,Mdga1,Ugt1a6b,Acsbg1,Adgrd1,Adgrf1,Angpt1,Apol6,Cdh15,Cracr2a,Cyp11a1,Doc2b,Eno1b,Ifitm1,Il1r2,Il1rl1,Il18rap,Lama3,Nptxr,Ocstamp,Prickle1,Ptpn3,Ryr3,Selenbp2,Prss56,Slc27a3,Slc6a4,Stra6,Syt13,Sytl2,Tmem158,Tmem63c,Tnfsfm13*	0.035
	extracellular region	14	*Cx3cl1,Sspo,Angpt1,Apol6,Bmp5,Cpa6,Il1r2,Il1rl1,Lama3,Lcn2,Lum,Mdk,Tslp,Tg*	0.035
Molecular function	interleukin-1 receptor activity	3	*Il1r2,Il1rl1,Il18rap*	0.02

FDR (False Discover Rate) is the probability of the number of genes out of the total gene number in the GO term and KEGG list. The genes in each GO term and KEGG are compared with the background distribution of annotation. A lower FDR represents the association of significant GO terms and KEGG with the group of genes. FDR < 0.05 indicates a significant association. No significant expression in biological process category and KEGG pathway in downregulation.

Table S5. Up-regulation and down-regulation of identical gene in both M2/S1 and M2/S2.

**Gene**	**logFC**	**FDR**
**Upregulation**		
Hspa1b[#]	1.09731	0.000000415
Gm14430	1.14529	0.000000007
Gzme	1.1463	0.000187673
Pbp2	1.2543	0.023008379
Gzmd	1.26245	0.000000067
Dnah10	1.40495	0.000012009
Fam110c	1.43956	0.000001383
Armcx4	1.6613	0.000000000
Wfdc1	1.85458	0.000025020
Gzmg	1.88187	0.000000000
Gap43	1.9311	0.003675370
Cep126	2.10367	0.000009413
Plscr2	2.10925	0.000000022
Ica1l	2.36078	0.000154729
Gm17757	7.81255	0.000000000
**Downregulation**		
Amd1	-12.4936	0.000000000
Gm18853	-11.2185	0.000000000

Hba-a2	-8.55152	0.000000000
Ugt1a6b	-8.00603	0.000000059
Eno1b	-7.77703	0.000000833
Ttn	-2.40002	0.000000000
Gvin1	-2.24451	0.000000000
Gm4724	-1.94475	0.000000000
Cyp11a1	-1.79065	0.022343340
Lama3	-1.74262	0.000000000
Cdh15	-1.45589	0.040536993
Gad1[#]	-1.45133	0.018940898
Ifitm1	-1.38069	0.000001103
Apol6	-1.35299	0.000000033
Adgrd1	-1.32604	0.010947496
Angpt1	-1.30098	0.004191883
Lcn2	-1.29107	0.000431621
Doc2b	-1.2238	0.040338784
Slc27a3	-1.21233	0.000417543
Nptxr	-1.18881	0.000000010
Rbms3	-1.17299	0.000011581
Sspo	-1.14053	0.000002138
Tnfsfm13	-1.08435	0.007492529
Syt13	-1.05443	0.000360497

Mrvi1#	-1.01635	0.031464125
Pkp1	-1.01538	0.001000188
Gm14308	-1.00886	0.000032119
Cd1d1#	-1.00855	0.000408275

The identical hub genes showed in KEGG pathways of M2/S1.
